# Time Trends in Survival After Surgery for Esophageal Cancer in a National Population-Based Study in Sweden

**DOI:** 10.1245/s10434-025-17007-3

**Published:** 2025-02-17

**Authors:** Ellinor Lundberg, Fredrik Mattsson, Eivind Gottlieb-Vedi, Jesper Lagergren

**Affiliations:** 1 Upper Gastrointestinal Surgery, Department of Molecular Medicine and Surgery, Karolinska Institutet, Karolinska University Hospital, Stockholm, Sweden; 2 School of Cancer and Pharmaceutical Sciences, King’s College London, London, UK

**Keywords:** Esophageal neoplasm, Esophageal cancer, Adenocarcinoma, Squamous cell carcinoma, Mortality

## Abstract

**Background:**

The long-term survival after surgery for esophageal cancer has improved over the past few decades, but studies that assess recent survival trends are lacking.

**Methods:**

This population-based cohort study included 2291 patients who underwent esophagectomy for esophageal cancer in Sweden between 2000 and 2020, with follow-up until 2024. Data came from medical records and national registries. Calendar time was analyzed as a continuous and categorized variable. The main outcome was all-cause 5-year mortality. Secondary outcomes were disease-specific 5-year mortality and 1-year all-cause mortality. Multivariable Cox regression provided hazard ratios (HR) with 95% confidence intervals (CI), adjusted for age, sex, comorbidity, tumor histology, neoadjuvant therapy, hospital volume, and pathological tumor stage.

**Results:**

The study period witnessed increasing resection rates, centralization to fewer hospitals, and improving postoperative 5-year survival. When analyzing calendar time as a continuous variable, the adjusted HR for all-cause 5-year mortality was 0.97 (95% CI 0.95–0.98). In categorized analyses, the HRs decreased for each later time period and was 0.57 (95% CI 0.47–0.69) comparing the surgery period 2015–2020 with 2000–2004. The trends were similar for disease-specific 5-year mortality and all-cause 1-year mortality. In stratified analyses, patients with Charlson comorbidity score ≥2 had the strongest improvement in all-cause 5-year mortality (HR 0.45, 95% CI 0.30–0.69 comparing surgery in 2015–2020 with 2000–2004).

**Conclusions:**

The recent 5-year survival has improved after surgery for esophageal cancer in Sweden. This improvement is not explained by lower surgery rates or selection of surgical candidates of younger age, fewer comorbidities, or earlier tumor stage.

Esophageal cancer ranks seventh globally in terms of cancer-related deaths and is the 11th most common cancer diagnosis.^1^ The overall survival in esophageal cancer has improved in most populations over the past decades, which is probably due to advancements in tumor detection, patient selection, and treatment.^1^ The main curative treatment is surgical resection, i.e., esophagectomy, often combined with neoadjuvant or perioperative chemotherapy or chemoradiotherapy.^2^ Because of advanced tumor stages or unsuitability, most patients with esophageal cancer are not eligible for an esophagectomy, and only a small number of individuals have early-stage cancer that can be removed endoscopically.^3^

Over the past few decades, many nations, including Sweden, have seen increases in the 5-year survival rate following curative treatment for esophageal cancer. Unselected (population-based) cohorts from a decade ago have estimated the 5-year survival at 30–40%.^4–8^ No population-based studies have assessed more recent time periods in survival, and the mechanisms explaining the time trends in survival are insufficiently understood. We have recently retrieved and reviewed comprehensive data from all patients who underwent esophagectomy for esophageal cancer in Sweden until the end of 2020 with follow-up for mortality until 2024, which provides opportunities to cover these knowledge gaps.

The goals of this study were to assess the recent survival trends of patients who have undergone surgery for esophageal cancer in a population-based setting and also to reveal explanations for the trends, including the role of resection rates, prognostic factors, patient selection, centralization of surgery, and neoadjuvant therapy.

## Methods

### Design

This population-based cohort study examined time trends in survival after esophagectomy for esophageal cancer, including cardia adenocarcinoma Siewert type I and II, in Sweden from 2000 until the end of 2020, with follow-up until May 15, 2024. The main outcome was all-cause 5-year mortality, and secondary outcomes were disease-specific 5-year mortality (defined by a diagnosis of esophageal or gastric cancer in the Cause of Death Registry) and all-cause 1-year mortality. The study was approved by the Regional Ethical Review Board in Stockholm.

### Source Cohort

We used data from the Swedish Esophageal Cancer Surgery Study (SESS), which is a cohort of almost all patients (98%) in Sweden who have undergone surgery for esophageal cancer since 1987. Data for SESS came from a comprehensive review of medical records combined with national Swedish health data registries of almost 100% completeness. The specific registries used for the present study were the nationwide Cancer Registry, Patient Registry, and Cause of Death Registry, which all have data of high validity and 100% or near 100% completeness.^9–11^ Earlier versions of SESS have been described elsewhere.^12–15^ This study was based on the most recently updated version of SESS, which added patients who underwent esophagectomy during the 5-year period from 2016 to 2020 and extended the follow-up for mortality until May 15, 2024. Race/ethnicity data were not available.

### Statistical Analysis

Changes in survival during the study period were analyzed as a continuous variable (for each calendar year) and also categorized into four calendar periods: 2000–2004, 2005–2009, 2010–2014, and 2015–2020. Crude survival curves comparing the four calendar periods were depicted by using the Kaplan-Meier method. Cox regression was used to calculate hazard ratios (HR) with 95% confidence intervals (CI) of the association between the exposure year of operation and risk of mortality. We used a crude model without any adjustments and a main model with multivariable adjustments for seven variables: age (continuous), sex (male or female), comorbidity (Charlson comorbidity index score 0, 1, or ≥2, not counting esophageal or gastric cancer), tumor histology (adenocarcinoma or squamous cell carcinoma), neoadjuvant therapy (yes or no), hospital volume of esophagectomy (using a moving 4-year period before the year of surgery for each patient), and pathological tumor stage (0–I, II, III, or IV). We used the most well-validated version of the Charlson comorbidity index with a cutoff of 10 years ago to assess comorbidities.^16,17^ For tumor stage, we used the eighth version of the TNM Classification of Malignant Tumours by the Union for International Cancer Control (UICC). Patients with partially missing data on any variable were few (2.6%) and thus were excluded from the study to allow the complete case analysis strategy. Effect modification was evaluated for each of the seven variables above, but using fewer categories in some variables to retain statistical power, by including an interaction term in the main multivariable model one by one. Thereafter, the HRs were derived within each stratum. The likelihood ratio test was performed, which indicated no significant interaction terms on significance level of alpha 0.05. The proportional hazards assumption was evaluated by log–log survival plots and by calculating the correlations between Schoenfeld residuals for a particular covariate and ranking of individual failure time. The correlations were low, indicating that the proportional hazards assumption was met for all covariates. The analyses followed a predefined study protocol and were led by an experienced biostatistician (FM) who used the statistical software SAS, Version 9.4 (SAS Institute Inc., Cary, NC).

## Results

### Patients

**Fig. 1:**
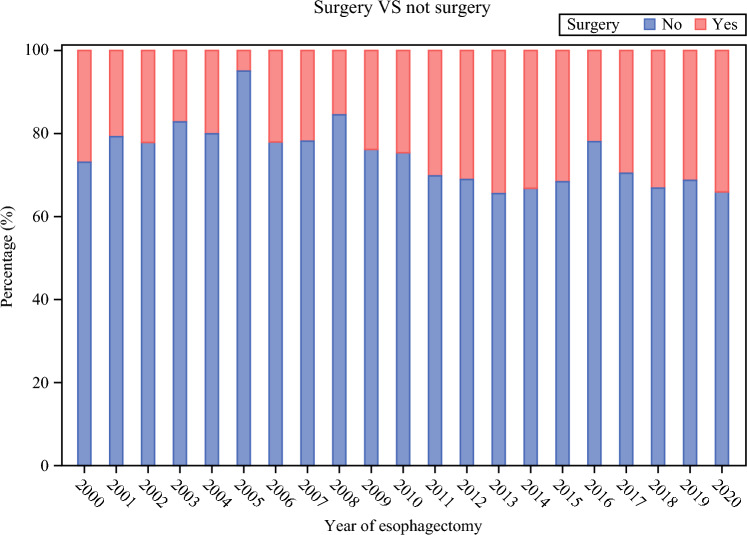
Proportion of patients who underwent esophagectomy with esophageal cancer in Sweden between 2000 and 2020

**Fig. 2:**
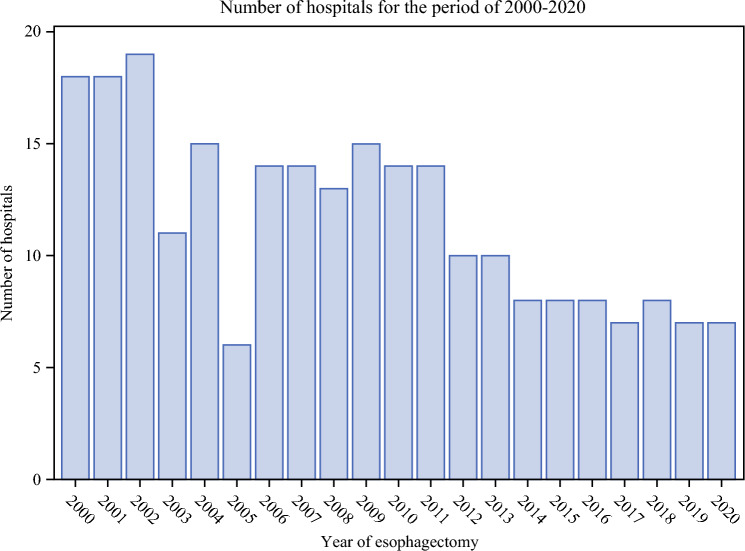
Number of hospitals performing esophagectomy for esophageal cancer in Sweden between 2000 and 2020

**Fig. 3:**
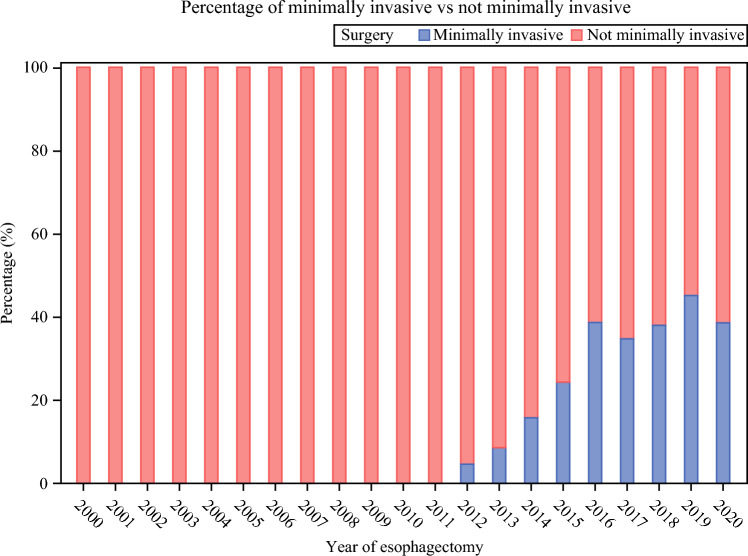
Proportion of patients who underwent minimally invasive esophagectomy for esophageal cancer in Sweden between 2000 and 2020

The study included 2,291 patients who underwent esophagectomy for esophageal cancer in Sweden between the years 2000 and 2020 with complete data on all variables. Over the study period, the number of operated patients increased (Table 1), the resection rates increased (Fig. 1), the surgery was conducted in fewer hospitals (Fig. 2), and the adoption of minimally invasive esophagectomy started and increased (Fig. 3). Characteristics of the patients, grouped into the four calendar periods, are presented in Table 1. There were no major differences in age or sex distribution between the calendar year groups, although the median age was highest in the most recent period (2015–2020). There were increasing rates of patients with more comorbidities (Charlson comorbidity score ≥2), adenocarcinoma histology, neoadjuvant therapy, and tumor stage IV during the study period, and the hospital volume of esophagectomy increased for each later calendar period (Table 1).

**Table 1:** Characteristics of 2291 patients who underwent esophagectomy for esophageal cancer in Sweden between 2000 and 2020

	Year 2000–2004	Year 2005–2009	Year 2010–2014	Year 2015–2020
Total	393 (17.5)	338 (14.8)	672 (29.3)	888 (38.8)
Age, median (interquartile range)	66.2 (58.3–72.7)	63.9 (59.3–70.9)	67.2 (61.0–72.7)	68.6 (61.8–73.8)
Sex				
Male	300 (76.3)	268 (79.3)	540 (80.4)	715 (80.5)
Female	93 (23.7)	70 (20.7)	132 (19.6)	173 (19.5)
Charlson comorbidity score				
0	246 (62.6)	175 (51.8)	359 (53.4)	460 (51.8)
1	107 (27.2)	105 (31.1)	216 (32.1)	254 (28.6)
≥2	40 (10.2)	58 (17.2)	97 (14.4)	174 (19.6)
Tumor histology				
Adenocarcinoma	221 (56.2)	195 (57.7)	516 (76.8)	731 (82.3)
Squamous cell carcinoma	172 (43.8)	143 (42.3)	156 (23.2)	157 (17.7)
Neoadjuvant treatment				
Yes	57 (14.5)	149 (44.1)	434 (64.6)	715 (80.5)
No	336 (85.5)	189 (55.9)	238 (35.4)	173 (19.5)
Hospital volume, median (interquartile range)	9.5 (3.8–18.0)	11.5 (5.3–13.3)	16.0 (9.5–25.5)	24.0 (16.3–38.0)
Pathological tumor stage				
0–I	89 (22.7)	107 (31.7)	239 (35.6)	304 (34.2)
II	123 (31.3)	122 (36.1)	117 (17.4)	152 (17.1)
III	150 (38.2)	89 (26.3)	211 (31.4)	281 (31.6)
IV	31 (7.9)	20 (5.9)	105 (15.6)	151 (17.0)
Deaths^a^				
All-cause 5-year mortality	279 (71.0)	211 (62.4)	401 (59.7)	501 (56.4)
Disease-specific 5-year mortality	257 (65.4)	188 (55.6)	358 (53.3)	428 (48.2)
All-cause 1-year mortality	131 (33.3)	97 (28.7)	160 (23.8)	203 (22.9)

Data are numbers with percentages in parentheses unless otherwise indicated

^a^Patients treated for esophageal cancer in 2019–2020 did not have a complete 5-year follow-up

### Trends in Absolute Survival

**Fig. 4:**
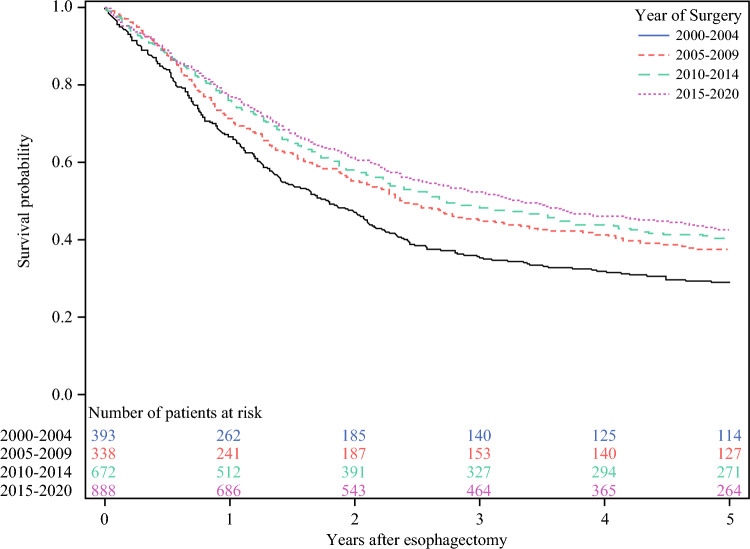
Kaplan-Meier survival curves after esophagectomy for esophageal cancer in Sweden by four calendar periods between 2000 and 2020

The all-cause 5-year mortality, disease-specific 5-year mortality, and all-cause 1-year mortality rates decreased for each later calendar period of surgery (Table 1). The survival probability increased for each later calendar period (Fig. 4).

### Trends in Risk of Mortality with Calendar Time as a Continuous Variable

The Cox regression analyses of calendar time as a continuous variable are presented in Table 2. Both the crude and the adjusted models showed decreasing HRs of mortality over the study period. For all-cause 5-year mortality, the HRs were similar in the crude and adjusted model, whereas the point estimates for disease-specific 5-year mortality and all-cause 1-year mortality were further decreased in the adjusted model compared to the crude model. The adjusted HRs were 0.97 (95% CI 0.95–0.98) for all-cause 5-year mortality, 0.96 (95% CI 0.95–0.97) for disease-specific 5-year mortality, and 0.96 (95% CI 0.95–0.98) for all-cause 1-year mortality.

**Table 2:** Risk of mortality after esophagectomy for esophageal cancer in Sweden between 2000 and 2020, with calendar time analyzed as a continuous variable

Outcome	Crude hazard ratio (95% confidence interval)	Adjusted hazard ratio (95% confidence interval)^a^
All-cause 5-year mortality	0.98 (0.97–0.99)	0.97 (0.95–0.98)
Disease-specific 5-year mortality	0.97 (0.96–0.98)	0.96 (0.95–0.97)
All-cause 1-year mortality	0.98 (0.96–0.99)	0.96 (0.95–0.98)

^a^Adjusted for age, sex, comorbidity, tumor histology, neoadjuvant therapy, hospital volume of esophagectomy, and pathological tumor stage

### Trends in Risk of Mortality with Calendar Time as a Categorical Variable

In the analyses of calendar time as a categorical variable, the HRs of all mortality outcomes decreased for each later calendar period (Table 3). The adjusted HRs tended to be more decreased than the crude HRs. Comparing the last time period (2015–2020) with the first (2000–004) showed adjusted HRs of 0.57 (95% CI 0.47–0.69) for all-cause 5-year mortality, 0.51 (95% CI 0.41–0.63) for disease-specific 5-year mortality, and 0.52 (95% CI 0.38–0.70) for all-cause 1-year mortality. Improvements in survival over calendar periods were also seen in the stratified analyses examining the risk of 5-year all-cause mortality in the adjusted model (Table 4). The point estimates indicated stronger improvements in survival in patients of older age, female sex, higher comorbidity scores, adenocarcinoma histology, no neoadjuvant therapy, surgery conducted at hospitals of higher volume, and more advanced tumor stages. However, none of these potential differences were statistically significant, as identified from the overlapping confidence intervals. The single lowest HR was found in patients with Charlson comorbidity score ≥2 (HR 0.45, 95% CI 0.30–0.69, comparing surgery during the calendar period 2015–2020 with 2000–2004).

**Table 3:** Risk of mortality after esophagectomy for esophageal cancer in Sweden between 2000 and 2020, with calendar time as a categorical variable

Outcome	Calendar period	Crude hazard ratio (95% confidence interval)	Adjusted hazard ratio (95% confidence interval)^a^
All-cause 5-year mortality	2000–2004	1.00 (reference)	1.00 (reference)
	2005–2009	0.78 (0.65–0.93)	0.79 (0.65–0.95)
	2010–2014	0.71 (0.61–0.83)	0.64 (0.54–0.77)
	2015–2020	0.67 (0.58–0.77)	0.57 (0.47–0.69)
Disease-specific 5-year mortality	2000–2004	1.00 (reference)	1.00 (reference)
	2005–2009	0.76 (0.63–0.91)	0.77 (0.64–0.94)
	2010–2014	0.70 (0.59–0.82)	0.61 (0.51–0.74)
	2015–2020	0.62 (0.53–0.72)	0.51 (0.41–0.63)
All-cause 1-year mortality	*2000–2004*	*1.00 (reference)*	1.00 (reference)
	*2005–2009*	*0.82 (0.63–1.06)*	0.82 (0.63–1.08)
	*2010–2014*	*0.67 (0.53–0.84)*	0.58 (0.44–0.75)
	*2015–2020*	*0.64 (0.51–0.80)*	0.52 (0.38–0.70)

^a^Adjusted for age, sex, comorbidity, tumor histology, neoadjuvant therapy, hospital volume of esophagectomy, and pathological tumor stage

**Table 4:** Risk of all-cause 5-year mortality after esophagectomy for esophageal cancer in Sweden between 2000 and 2020, stratified by covariates

Covariate	Year 2000–2004	Year 2005–2009	Year 2010–2014	Year 2015–2020
	Hazard ratio (95% confidence interval)^a^	Hazard ratio (95% confidence interval)^a^	Hazard ratio (95% confidence interval)^a^	Hazard ratio (95% confidence interval)^a^
Age				
Quartile 1–2	1.00 (reference)	0.85 (0.66–1.01)	0.65 (0.51–0.83)	0.64 (0.49–0.83)
Quartile 3–4 (including the median)	1.00 (reference)	0.75 (0.56–0.98)	0.67 (0.54–0.84)	0.56 (0.44–0.71)
Sex				
Male	1.00 (reference)	0.82 (0.67–1.01)	0.69 (0.57–0.83)	0.59 (0.48–0.73)
Female	1.00 (reference)	0.68 (0.46–1.01)	0.47 (0.32–0.68)	0.50 (0.35–0.72)
Charlson comorbidity score				
0	1.00 (reference)	0.71 (0.55–0.91)	0.61 (0.49–0.76)	0.57 (0.45–0.73)
1	1.00 (reference)	0.88 (0.63–1.22)	0.78 (0.5–1.04)	0.65 (0.48–0.88)
≥2	1.00 (reference)	0.87 (0.55–1.38)	0.53 (0.35–0.82)	0.45 (0.30–0.69)
Tumor histology				
Adenocarcinoma	1.00 (reference)	0.70 (0.55–0.90)	0.61 (0.50–0.76)	0.56 (0.44–0.70)
Squamous cell carcinoma	1.00 (reference)	0.95 (0.73–1.25)	0.73 (0.55–0.96)	0.60 (0.44–0.82)
Neoadjuvant treatment				
Yes	1.00 (reference)	1.04 (0.71–1.52)	0.82 (0.58–1.16)	0.70 (0.49–0.99)
No	1.00 (reference)	0.74 (0.60–0.93)	0.61 (0.49–0.75)	0.61 (0.47–0.79)
Hospital volume				
Quartile 1–2	1.00 (reference)	0.76 (0.62–0.93)	0.62 (0.51–0.77)	0.60 (0.47–0.78)
Quartile 3–4 (including the median)	1.00 (reference)	1.10 (0.71–1.68)	0.69 (0.53–0.90)	0.57 (0.44–0.74)
Pathological tumor stage				
0–II	1.00 (reference)	0.94 (0.73–1.22)	0.66 (0.51–0.85)	0.62 (0.47–0.80)
III–IV	1.00 (reference)	0.72 (0.55–0.94)	0.64 (0.52–0.80)	0.55 (0.44–0.70)

^a^Adjusted for age, sex, comorbidity, tumor histology, neoadjuvant therapy, hospital volume of esophagectomy, and pathological tumor stage

## Discussion

This study displays that survival after surgery for esophageal cancer has continued to increased in Sweden in recent years, despite increasing resection rates and increasing rates of surgery on patients of older age, more comorbidity, and more advanced tumor stage.

Among strengths of this study is that we used recent data from an entire country with assessment of survival until 2024. The population-based design with almost complete inclusion of patients who underwent esophagectomy for esophageal cancer in Sweden during the study period minimized selection bias and facilitates generalizability of the results to countries with similar populations and healthcare. The richness in high-quality and well-validated variables enabled adjustment for all main prognostic factors, which counteracted confounding.^9–11^ Another advantage is the 100% complete follow-up of mortality in the Cause of Death Registry, which guaranteed no losses to follow-up.^11^ A limitation is that residual confounding cannot be ruled out in an observational study, although the results were adjusted for key variables. Despite the large cohort size, some of the subgroup analyses had limited statistical power. To lessen the possibility of errors resulting from multiple testing, we restricted the number of analyses.

In two earlier Swedish population-based studies (from our research group), the 5-year survival after surgery for esophageal cancer increased for each calendar year period from 19.7% in 1987–1991 to 30.7% in 1997–2000 but did not improve further in 2001–2005 (30.5%).^4,5^ In a later Swedish population-based study assessing survival until 2013, the postoperative relative 5-year survival continued to increase.^6^ Data from several other countries display similar positive trends. In a Spanish study, the postoperative 5-year survival rate in esophageal cancer increased from 43% in 2000–2007 to 64% in 2008–2015.^7^ A study from the Netherlands found that the 5-year survival rate after surgical treatment for esophageal cancer improved from 32.8 to 48.2% between 1993 and 2017.^8^

It has been argued that the improved long-term survival after surgery for esophageal cancer may be explained by a more restrictive approach to surgery, mirrored by higher rates of younger patients with better fitness and less advanced tumor stage, and lower resection rates overall.^18^ However, in this study the resection rate instead increased during the study period and older age, higher comorbidity scores and more advanced tumor stage were instead more frequent in the later calendar periods. Consequently, the adjustment for age, comorbidity, and tumor stage did not explain the decreased HRs found in the present study but rather strengthened them. These results imply that rather than the contrary, surgery was performed more often in more recent years on patients who were thought to have a worse prognosis. Thus, more conservative strategies for selecting patients for surgery do not explain the identified improvements in survival in the present study. Performing esophagectomies increasingly frequently on older patients with more comorbidities has also been shown to work well in other studies.^19^

Other reasons for the improved survival after esophagectomy may be the introduction of routine use of advanced imaging techniques prior to surgery, e.g., computerized tomography and positron emission tomography. This may have contributed to a more accurate staging and thus better selection of patients who benefit most from surgery.^20^ Another change during the study period is the increased use and advancements in neoadjuvant treatment, which may have contributed to the better survival rates.^21^ However, adjustment for neoadjuvant therapy did not change the findings. The standardization and centralization to fewer hospitals and surgeons may have contributed to the findings, although the adjustment for hospital volume did not influence the results.^13^ The shift toward minimally invasive techniques took place during the last part of the study period, which might have contributed to improved survival rates. A survival benefit of minimally invasive versus open esophagectomy was found in another study using an earlier version of this Swedish cohort.^22^ The developments in preoperative imaging, neoadjuvant therapies, and surgical strategy over the study period may have contributed to improved survival and should reduce the frequency of patients where resection was attempted, but failed, i.e., open-and-close cases open-and-close cases. There are likely a number of contributing factors to the higher survival rates following esophageal cancer surgery.

A point of discussion is how to further improve the selection of patients with esophageal cancer for surgery. This study shows a trend of operating on patients of older age, more comorbidity, and more advanced tumor stage, yet still providing better overall survival than before. Conversely, performing esophagectomies that result in mortality in a short period of time is a poor outcome, because patients seldom fully heal from the procedure before passing away. Finding the optimal balance in the selection process is of great relevance for both patients and society at large and is an important topic for research.

## Conclusions

This Swedish population-based study with complete follow-up and adjustment for key confounders shows that the postoperative 5-year survival of esophageal patients has continued to increase over the past few years. This may be due to several factors, including improvements in preoperative imaging and surgical and nonsurgical treatment.
